# High throughput protease profiling comprehensively defines active site specificity for thrombin and ADAMTS13

**DOI:** 10.1038/s41598-018-21021-9

**Published:** 2018-02-12

**Authors:** Colin A. Kretz, Kärt Tomberg, Alexander Van Esbroeck, Andrew Yee, David Ginsburg

**Affiliations:** 1 Department of Medicine, McMaster University and the Thrombosis and Atherosclerosis Research Institute, Hamilton, Ontario Canada; 2 Life Sciences Institute, University of Michigan, Ann Arbor, MI USA; 3 Department of Human Genetics, University of Michigan, Ann Arbor, MI USA; 4 Department of Electrical Engineering and Computer Science, University of Michigan, Ann Arbor, MI USA; 5 Howard Hughes Medical Institute and Departments of Internal Medicine and Pediatrics, University of Michigan, Ann Arbor, MI USA

## Abstract

We have combined random 6 amino acid substrate phage display with high throughput sequencing to comprehensively define the active site specificity of the serine protease thrombin and the metalloprotease ADAMTS13. The substrate motif for thrombin was determined by >6,700 cleaved peptides, and was highly concordant with previous studies. In contrast, ADAMTS13 cleaved only 96 peptides (out of >10^7^ sequences), with no apparent consensus motif. However, when the hexapeptide library was substituted into the P3-P3′ interval of VWF73, an exosite-engaging substrate of ADAMTS13, 1670 unique peptides were cleaved. ADAMTS13 exhibited a general preference for aliphatic amino acids throughout the P3-P3′ interval, except at P2 where Arg was tolerated. The cleaved peptides assembled into a motif dominated by P3 Leu, and bulky aliphatic residues at P1 and P1′. Overall, the P3-P2′ amino acid sequence of von Willebrand Factor appears optimally evolved for ADAMTS13 recognition. These data confirm the critical role of exosite engagement for substrates to gain access to the active site of ADAMTS13, and define the substrate recognition motif for ADAMTS13. Combining substrate phage display with high throughput sequencing is a powerful approach for comprehensively defining the active site specificity of proteases.

## Introduction

The specificity of a protease for its substrate(s) is dictated by complex interactions of exosites to capture and appropriately orient the substrate with the active site, which catalyzes peptide bond hydrolysis^1^. While some proteases are highly selective for residues surrounding the P1-P1′ scissile bond^2^, others are more promiscuous^3–5^. For serine proteases, the fit of a substrate into the active site is largely dictated by the interaction of the P1 residue of the substrate with the S1-specificity pocket of the protease^6^. Thrombin, the final effector serine protease in the coagulation system, exhibits strong preference for Arg at position P1, although Lys can substitute for some substrates^7^. In contrast, metalloproteases are generally considered to be less-selective for amino acid content near the cleavage site^8,9^. However, recent studies suggest that the matrix metalloprotease family exhibits a preference for P3 proline and aliphatic residues at P1′^10^. Understanding the amino acid sequences recognized by proteases is critical because it can lead to novel diagnostic tools and may contribute to the development pharmaceutical agents^1^.

ADAMTS13, a member of the metzincin family of metalloproteases, regulates the platelet-binding capacity of von Willebrand Factor (VWF) by proteolytic processing^11^. ADAMTS13 cleaves VWF when sufficient shear forces unfold the A2 domain, exposing the cryptic Tyr1605-Met1606 scissile bond and a number of exosite-binding domains^12–14^. Deficiency in ADAMTS13 causes thrombotic thrombocytopenia purpura (TTP), a disorder characterized by thrombocytopenia and hemolytic anemia caused by deposition of VWF-rich thrombi in the microcirculation^15^. Fragments of VWF, such as VWF73 (comprising Asp1596-Arg1668), have been used as biochemical tools to study ADAMTS13 in an *in vitro* setting and form the basis for clinical assays of ADAMTS13 activity^16^. However, the efficiency of cleavage declines rapidly with shorter VWF fragments^17^, suggesting an important role for exosite interactions in VWF cleavage by ADAMTS13^17–21^.

M13 filamentous substrate phage display is a useful technique for probing the substrate recognition determinants of proteases^7,22^. However, after several rounds of selection^23^, biases in phage amplification, infectivity, and prokaryotic protein expression can limit the number of informative clones isolated with this technique. Recent advances in high throughput DNA sequencing technology^24^ have enabled comprehensive analysis of every clone in the library following a single round of selection^25–29^. By coupling substrate phage display with high throughput sequencing, we recently characterized a comprehensive VWF73 mutagenesis library, and showed that substitutions within the P3-P2′ interval were among the most deleterious to proteolysis by ADAMTS13^30^.

To further characterize the active site specificity of ADAMTS13, we now report comprehensive protease specificity profiling by combining random 6 amino acid substrate phage display and high throughput sequencing. As proof-of-concept, we define the most comprehensive substrate specificity profile for thrombin to-date, confirming known requirements for Arg at P1, and revealing both positive and negative regulators of thrombin substrate recognition. The poor recognition of peptides by ADAMTS13 was expanded 17-fold when the library was inserted into the P3-P3′ residues of VWF73, revealing a broader substrate recognition potential for ADAMTS13 than previously appreciated. These data confirm the importance of exosite engagement for ADAMTS13 substrate recognition, and provide a detailed substrate recognition profile that may guide identification of novel substrates.

## Results

### Characterization of substrate phage display library

**Figure 1:**
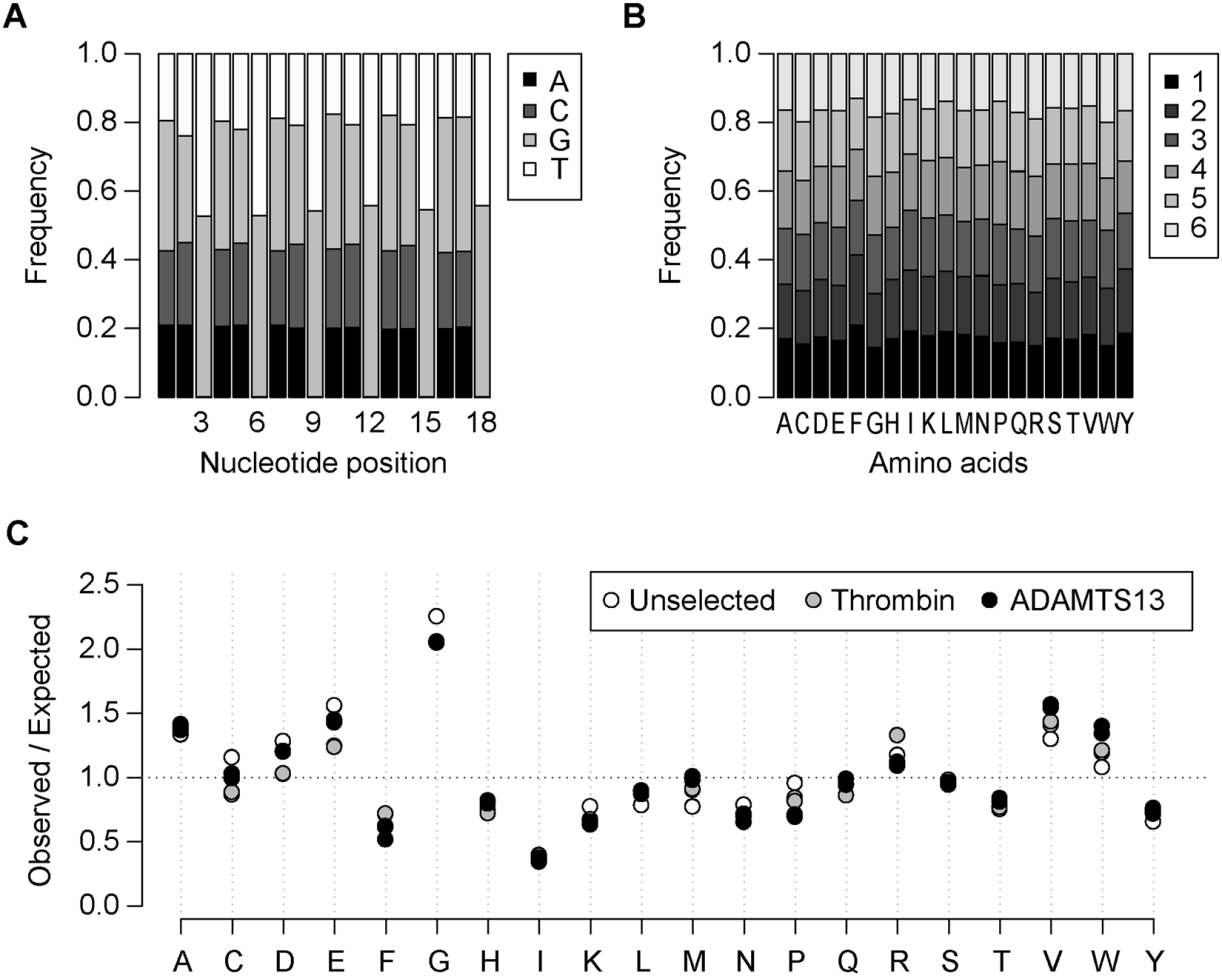
Nucleotide and Amino Acid distribution in NNK library. (**A**) The frequency of each nucleotide was calculated at all 18 positions within the unselected random 6 amino acid library. As expected, the first 2 positions of each codon contain all 4 nucleotides (N), in roughly equal proportions, while the third position contains only G and T (K). (**B**) The frequency of each amino acid was calculated for each position of the library. The representations for each amino acid across the 6 positions are roughly equivalent. TAG (91.8% of stops) was over-represented in the library compared to TAA (1.7% of stops) and TGA (6.5% of stops), consistent with NNK randomization. The presence of stop codons within the library suggests a mechanism to bypass stop codons at low levels during phage assembly, either through ribosomal read-through or alternative translation start sites. Because adenine should not be present in the third nucleotide position in the NNK randomization scheme, the lower representation of TAA and TGA compared to TAG likely represents background sequencing errors within the dataset. (**C**) The frequency of each amino acid was normalized to the number of codons in the NNK library for the unselected library (FLAG) and after selection of peptides cleaved by thrombin or ADAMTS13. In the unselected library and after both selections, the frequency of each amino acid varies from the frequency expected based on the number of codons for that amino acid in the NNK randomization scheme. The amino acid distribution within the unselected library likely reflects both codon usage for each amino acid as well as biased phage production for clones bearing peptides with particular amino acid content. The preference of either thrombin or ADAMTS13 for peptides with a specific amino acid content should also be observed as deviation from frequencies in the unselected library.

A random 6 amino acid substrate phage display library consisting of 2.3 × 10^8^ independent clones was constructed, which represents 3.5 X of the 20^6^ possible peptide sequences. High throughput sequencing of the unselected library confirmed the broad representation of sequences in the library (Figure S1, Fig. 1A) and revealed >5.5 million unique peptides (Table 1). More than 1 million peptides were identified by only a single sequencing read, likely a consequence of the library depth exceeding sequencing read depth. Each amino acid was comparably distributed across all 6 positions (Fig. 1B) with only modest deviation from expected frequencies (Fig. 1C). Stop codons should be limited in the FUSE55 phage display system because premature termination of the bacteriophage PIII protein abolishes phage assembly. Consistent with this prediction, only 0.04% of sequencing reads contained a stop codon, substantially lower than the 17% expected within the synthesized oligonucleotide.

**Table 1:** Random 6 amino acid peptide library.

Sample	Total reads	Passed filter (%)	Unique peptides	Mean count	Median count	Min-Max
Unselected	12,327,473	89.8	5,536,697	2.00	1	1–144
	13,296,736	89.7	5,791,291	2.06	1	1–170
Thrombin	12,334,072	89.7	5,324,398	2.08	1	1–254
	12,240,464	90.0	5,316,032	2.07	1	1–225
ADAMTS13	11,166,160	89.1	5,211,441	1.91	1	1–74
	12,076,701	89.9	5,379,005	2.02	1	1–71

The results of the high throughput sequencing data analysis pipeline are shown for two samples of the unselected random 6 amino acid peptide library, and following two selections of this library by thrombin and ADAMTS13. The table shows the total number of sequencing reads per sample (total reads), the percentage of reads that passed the quality filters (passed filter) and the total number of unique peptides that were ultimately identified (unique peptides). Also shown is the average number of sequencing counts for each unique peptide (mean count), the median number of counts for each peptide (median count), and the range of counts for each unique peptide (min-max).

### Thrombin Selection

**Figure 2:**
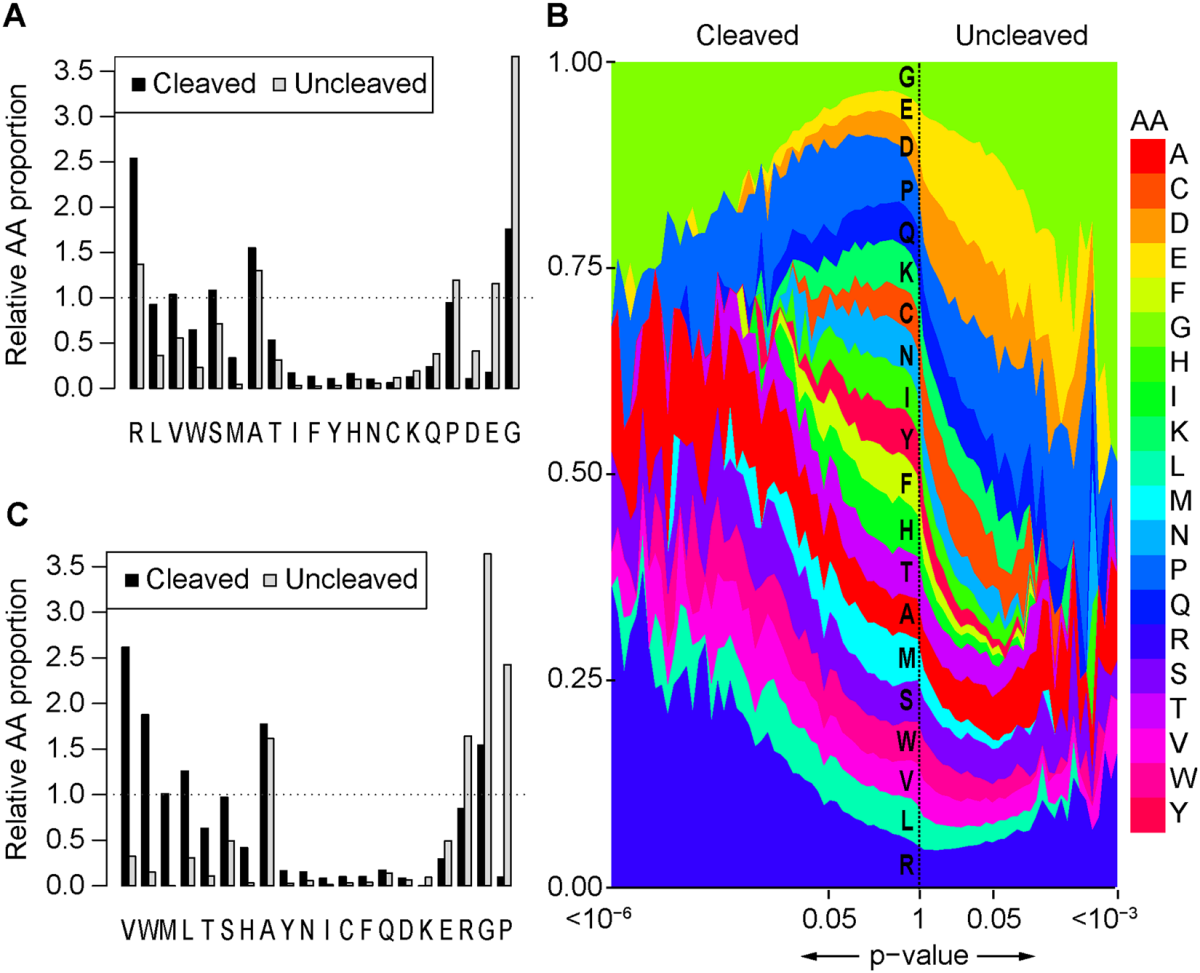
Amino acid enrichment and depletion for thrombin and ADAMTS13. (**A**) The relative proportion of each amino acid in the significantly cleaved or uncleaved peptides following thrombin selection compared to all peptides assessed in the experiment. Amino acids are sorted according to the difference in their relative proportion in cleaved peptides compared to uncleaved peptides. (**B**) The frequency of each amino acid was calculated for peptides grouped by p-value, with the most significantly enriched peptides on the left and most significantly depleted peptides on the right. Each data point is calculated as an average of frequencies for more than 10 peptides belonging to the same p-value subgroup on the logarithmic scale. Arg is the most abundant amino acid among the significantly enriched peptides, whereas Asp and Glu are the most abundant among the depleted peptides, and are not present at all among the enriched peptides. Gly is abundant in both enriched and depleted peptides, but is more abundant within the depleted peptides, suggesting a net-antagonistic role in thrombin substrate specificity. (**C**) Same as (**A**) but for ADAMTS13 selection.

To confirm the utility of high throughput sequencing to identify phage displaying cleavable peptides from a single round of selection, we screened the serine protease thrombin (Figure S2). Thrombin is a well-characterized serine protease, with known substrate recognition determinants. Out of 5.3 × 10^6^ unique peptide sequences identified following thrombin selection (Table 1, Figure S3), 6722 peptides were significantly enriched, and identified as cleaved (*p*_FDR_ < 0.05, Figure S4) (see Supplementary data 1). Analysis of selected phage sequences confirms a general preference for Arg and exclusion of acidic amino acids in the cleaved peptides (Fig. 2A). Arg was the dominant amino acid within the most significantly cleaved peptides (Fig. 2B), consistent with the known requirement at P1 of thrombin substrates^7^. Of the 18 cleaved peptides lacking Arg, 14 contained Lys.

**Figure 3:**
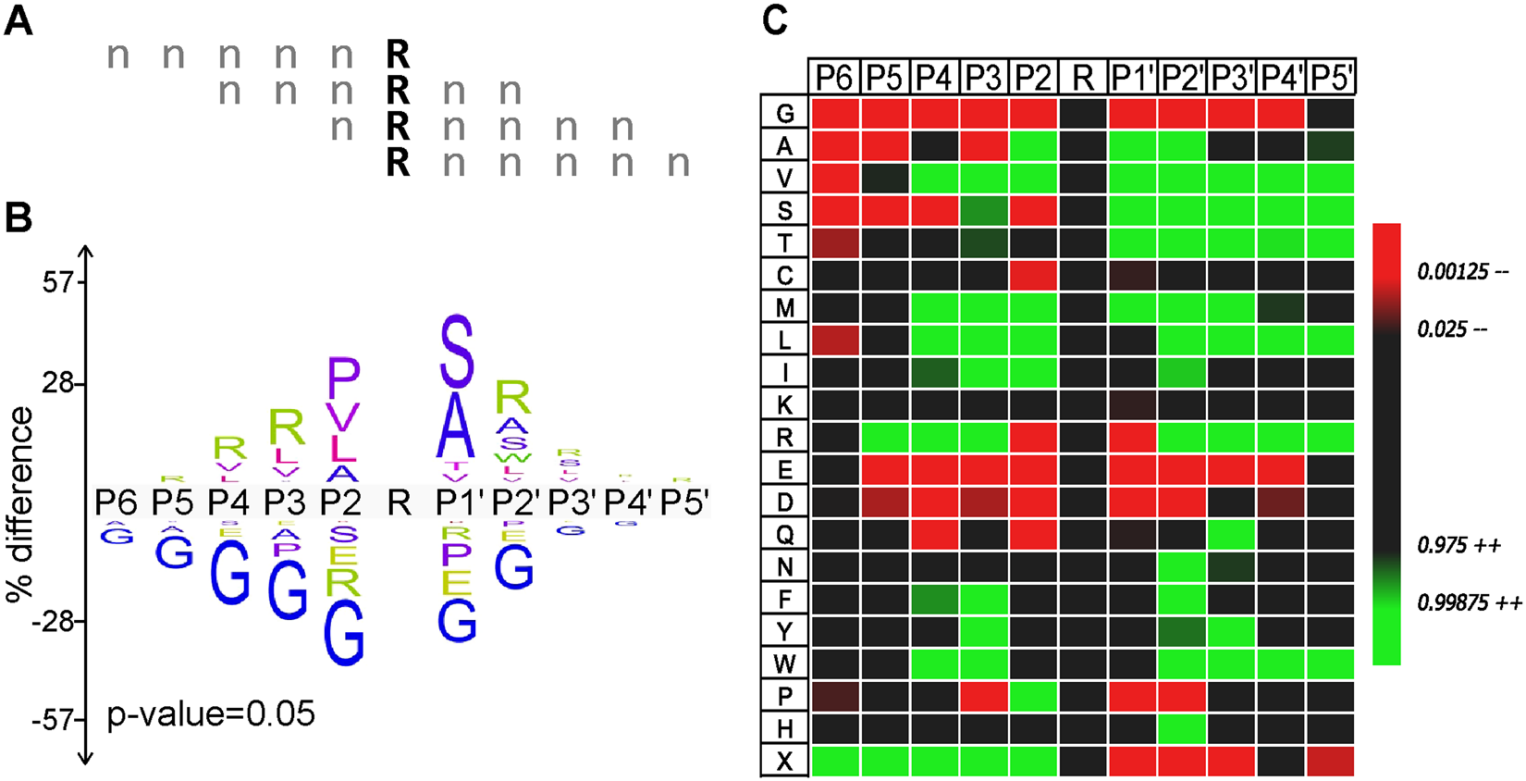
Thrombin Substrate motif. (**A**) Schematic overview for alignment of peptides assuming Arg as P1. For peptides containing multiple Arg residues, the center-most Arg was aligned, with preference given to position 3 Arg residues in peptides containing Arg residues at position 3 and 4. (**B**) The iceLogo plot representing the relative frequency of every amino acid in the cleaved (top) and uncleaved (bottom) peptide pools following thrombin selection. (**C**) The iceLogo heatmap shows the preference for each amino acid at every position in the cleaved (green) or uncleaved (red) peptide pools. Residues at positions that do not appreciably populate either pool are also indicated (black).

Although thrombin shows preference for Arg, 906/1992 significantly depleted peptides (uncleaved) contained at least one Arg residue. To determine amino acid motifs that promote or antagonize thrombin cleavage at Arg, all peptides containing an Arg in the cleaved and uncleaved peptide pools were aligned by assigning the Arg as P1 (see Methods) and compared (Fig. 3A). Low molecular weight amino acids at the presumptive P2 and P1′ positions promoted cleavage, with P2 Pro and P1′ Ser the dominant residues (Fig. 3B). In contrast, bulky aliphatic amino acids at P2 or P1′ antagonized cleavage, but promoted cleavage when present at more distal sites (Fig. 3C). By contrast, acidic and basic amino acids throughout the peptide antagonized thrombin cleavage. Analysis of peptides containing multiple Arg residues indicated that Arg at presumptive position P2 and/or P1′ antagonize thrombin substrate recognition (Fig. 3B). Analysis of cleaved and uncleaved peptides containing only a single Arg residue (Figure S5) yielded comparable results, suggesting that multiple Arg residues within a single peptide did not appreciably confound data analysis.

### ADAMTS13 Selection

Compared with thrombin, ADAMTS13 appears to exhibit narrow substrate specificity, since VWF is its only known substrate^11^. Consistent with this observation, only 96 cleaved peptides were identified from the random peptide library following overnight selection by ADAMTS13 (*p*_FDR_ < 0.05, Figure S4). Although cleaved peptides preferentially contained bulky hydrophobic amino acids (Fig. 2C), no obvious motif was observed (Figure S6), consistent with previous studies that demonstrate poor recognition of short peptidyl substrates by ADAMTS13^17,21^.

### VWF73(P3-P3′) selection by ADAMTS13

To address the role of exosite interactions in ADAMTS13 substrate recognition, the P3-P3′ residues within VWF73 were replaced with random amino acids. The VWF73(P3-P3′) library contained ~2.5 × 10^7^ independent clones, and high throughput sequencing showed the expected nucleotide composition (Figure S7), although amino acid frequencies deviated from expected (Figure S8), likely reflecting biases in displayed peptides due to phage production.

**Figure 4:**
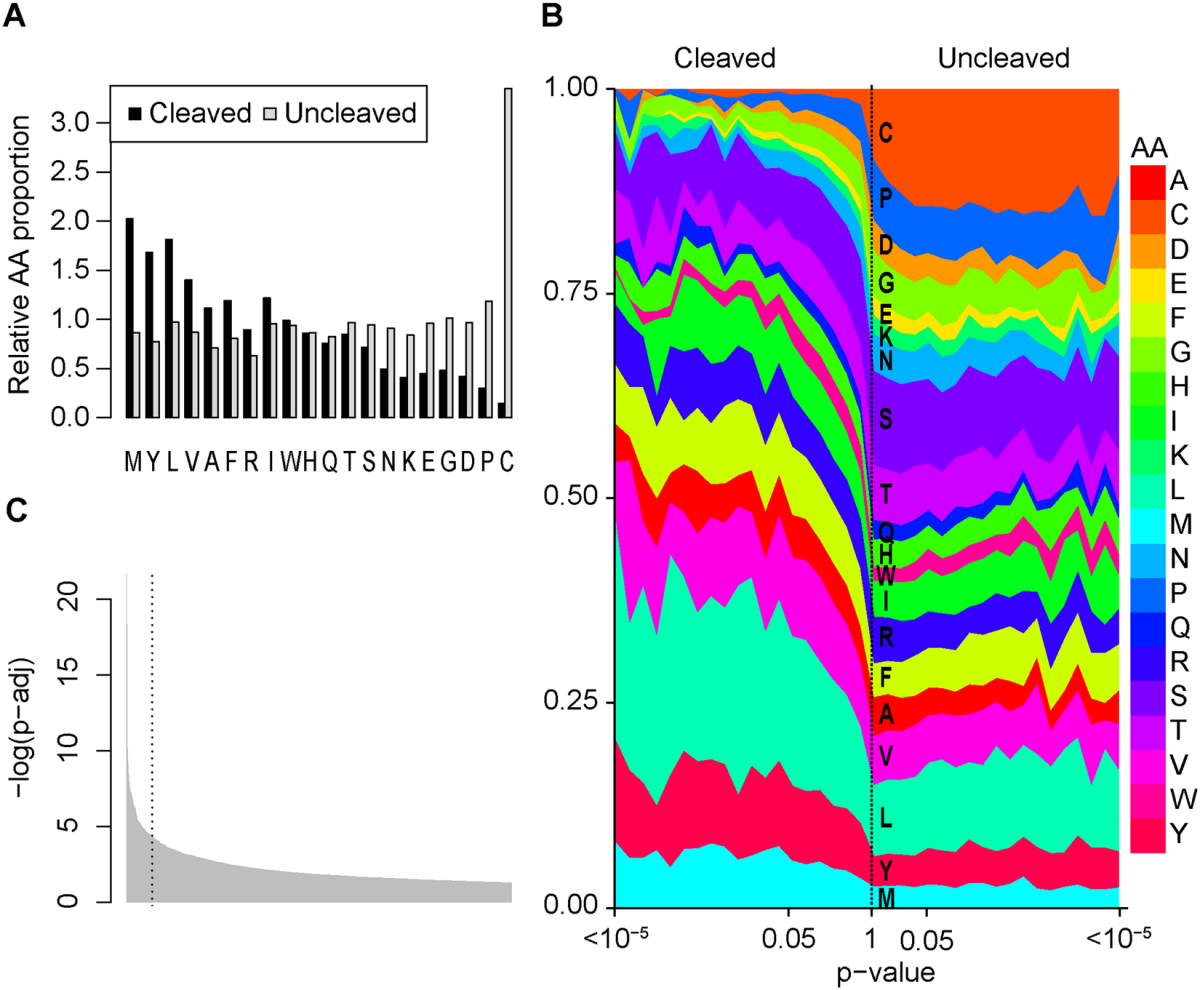
ADAMTS13 selection of VWF73(P3-P3′) random peptide library. (**A**) The relative proportion of each amino acid from significantly cleaved or uncleaved peptides following ADAMTS13 selection of the VWF73(P3-P3′) library compared to all unique peptides. (**B**) The frequency of each amino acid was calculated for peptides grouped by p-value, with the most significantly cleaved peptides on the left and most significantly uncleaved peptides on the right. Each data point is calculated as an average of frequencies for more than 10 peptides belonging to the same p-value subgroup on the logarithmic scale. (**C**) Rank-order p-value of all significantly cleaved peptides following VWF73(P3-P3′) selection by ADAMTS13. The P3-P3′ residues of native VWF (Leu-Val-Tyr-Met-Val-Trp), dashed line, was among the most enriched peptides.

Following treatment of the VWF73(P3-P3′) library with ADAMTS13, 1670 cleaved peptides were detected (*p*_FDR_ < 0.05, Supplementary data 2). Overall, bulky aliphatic amino acids were preferred in the enriched peptide pool, whereas acidic amino acids, as well as proline and cysteine, appeared to antagonize ADAMST13 substrate recognition (Fig. 4A,B). The native amino acid sequence for VWF within this interval (Leu-Val-Tyr-Met-Val-Thr) was among the top peptides identified (*p*_FDR_ = 8 × 10^−5^) (Fig. 4C), and none of the most significantly cleaved peptides exhibited faster substrate performance than wild type VWF73 (Table 2). As a result, peptides with lower P-values than wild type VWF73 are not necessarily cleaved more efficiently.

**Table 2:** Kinetic characterization of VWF73 library peptides.

VWF73 P3-P3′ peptide	Fold-change (log_2_)	k_cat_/K_M_ (×10^5^ M^−1^ min^−1^)
LVYMVT (WT)	0.84	300
LELYLS	0.49	0.15
IQLFLA	0.70	0.54
RLRYFL	0.79	1.74
IMMFLG	0.65	3.18
LRYSSM	0.66	1.44
LGLEHS	−1.20	No cleavage
LSVYGS	−1.09	No cleavage
NLQLIF	−1.79	No cleavage
SSWWMC	−1.76	No cleavage
APPVDS	−1.68	No cleavage

The top-ranked peptides from the VWF73(P3-P3′) library following selection by ADAMTS13 were identified from the most significantly enriched (Log2 fold change >0) or most significantly depleted (Log2 fold change <0) based on adjusted P-value. Phage were cloned with wild type VWF73 P3-P3′ residues replaced with the residues listed. Each individual phage clone was reacted with ADAMTS13 and cleavage was monitored at various reaction time points using AlphaLISA as previously described^30,53^. Individual k_cat_/K_M_ values were calculated for clones exhibiting detectable proteolysis by ADAMTS13. Clones with no detectable proteolysis are indicated as ‘no cleavage’.

**Figure 5:**
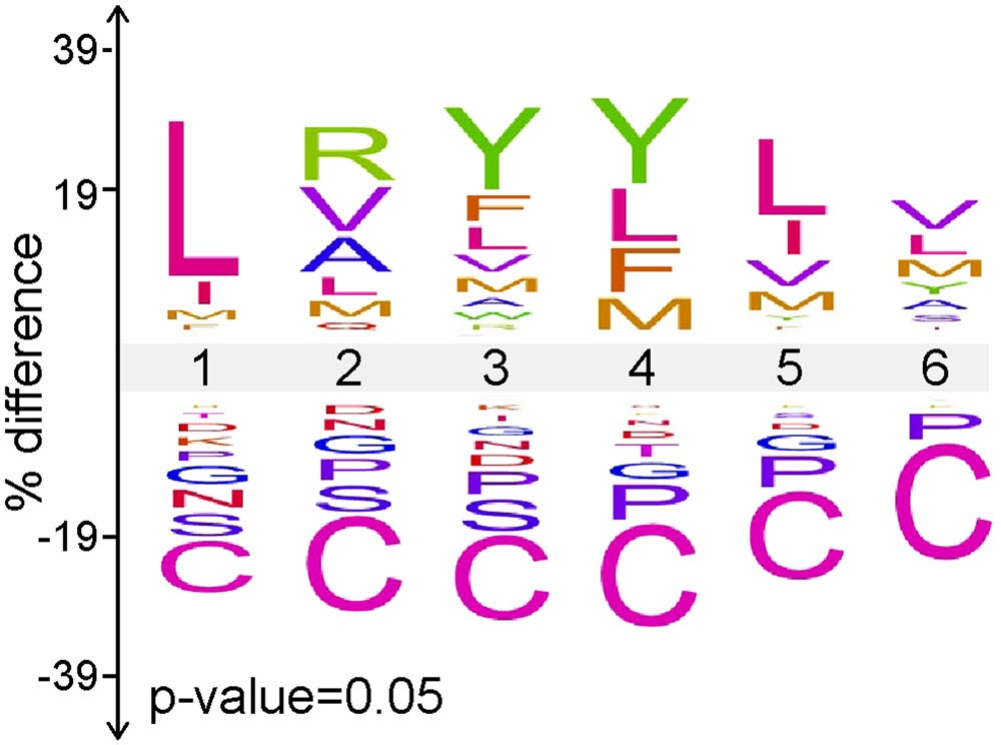
ADAMTS13 substrate recognition motif. The iceLogo plot representing the relative frequency of every amino acid in the cleaved (top) and uncleaved (bottom) peptide pools following ADAMTS13 selection of the VWF73(P3-P3′) library.

**Figure 6:**
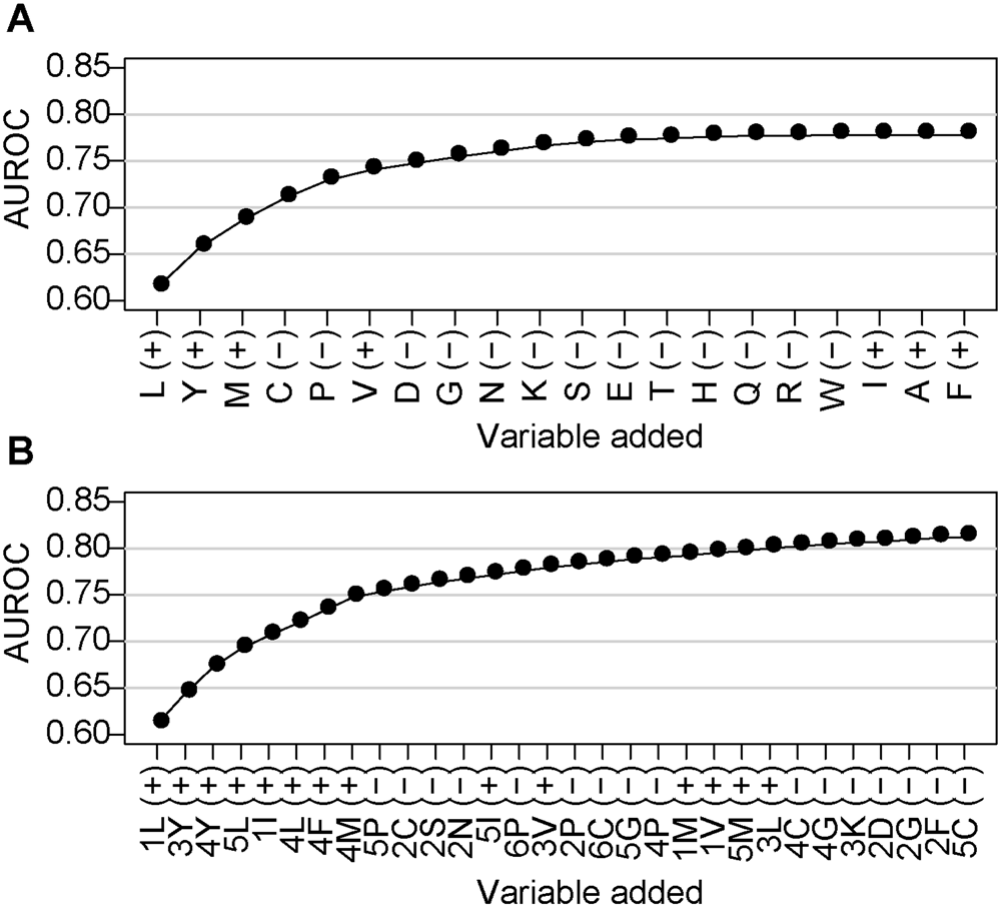
AUROC of the ADAMTS13 substrate motif. Logistic regression with forward stepwise feature selection was applied to ADAMTS13 selection of the VWF73(P3-P3′) library, with significantly cleaved peptides trained against neither significantly cleaved nor significantly depleted. This analysis provided a measure of which amino acid requirements were most useful for predicting ADAMTS13 substrate recognition, and how well a model based on the presence of these amino acids predicted peptide cleavage. Outcomes were iterated for amino acid regardless of position (**A**) or amino acids constrained to position (**B**). Both positive (+) and negative (−) regulators of ADAMTS13 substrate recognition were identified. Amino acid content alone yielded an Area Under Receiver Operating Curve (AUROC) of ~0.78 (**A**), whereas amino acid content at defined positions yielded a maximum AUROC of 0.8 (**B**).

Comparing the cleaved and uncleaved VWF73(P3-P3′) peptides reveals a coherent ADAMTS13 substrate recognition motif. At 5 out of 6 positions, the corresponding residue in VWF is among the most significantly enriched amino acids (Fig. 5). Approximately 75% of cleaved peptides contained Leu, with 34% containing Leu at amino acid position 1. Hierarchical cluster analysis of cleaved peptides trained against uncleaved peptides indicates a general preference for bulky aliphatic residues and exclusion of electrostatic amino acids and proline (Fig. 6A). Specifically, (Leu/Ile)1, Tyr3, (Leu/Tyr/Met/Phe)4 provided 75% of the predictive capacity for ADAMTS13 substrate recognition, relative to a randomly selected uncleaved sample (Fig. 6B), indicating their dominant roles in substrate recognition by ADAMTS13. In wild type VWF73, position 4 corresponds to the P1′ residue. This residue has previously been shown to be critical for metalloprotease substrate recognition^31–33^, consistent with the dominant feature for bulky aliphatic residues at position 4. Overall, these data suggest a substrate specificity profile for ADAMTS13 largely dictated by bulky aliphatic amino acids.

**Figure 7:**
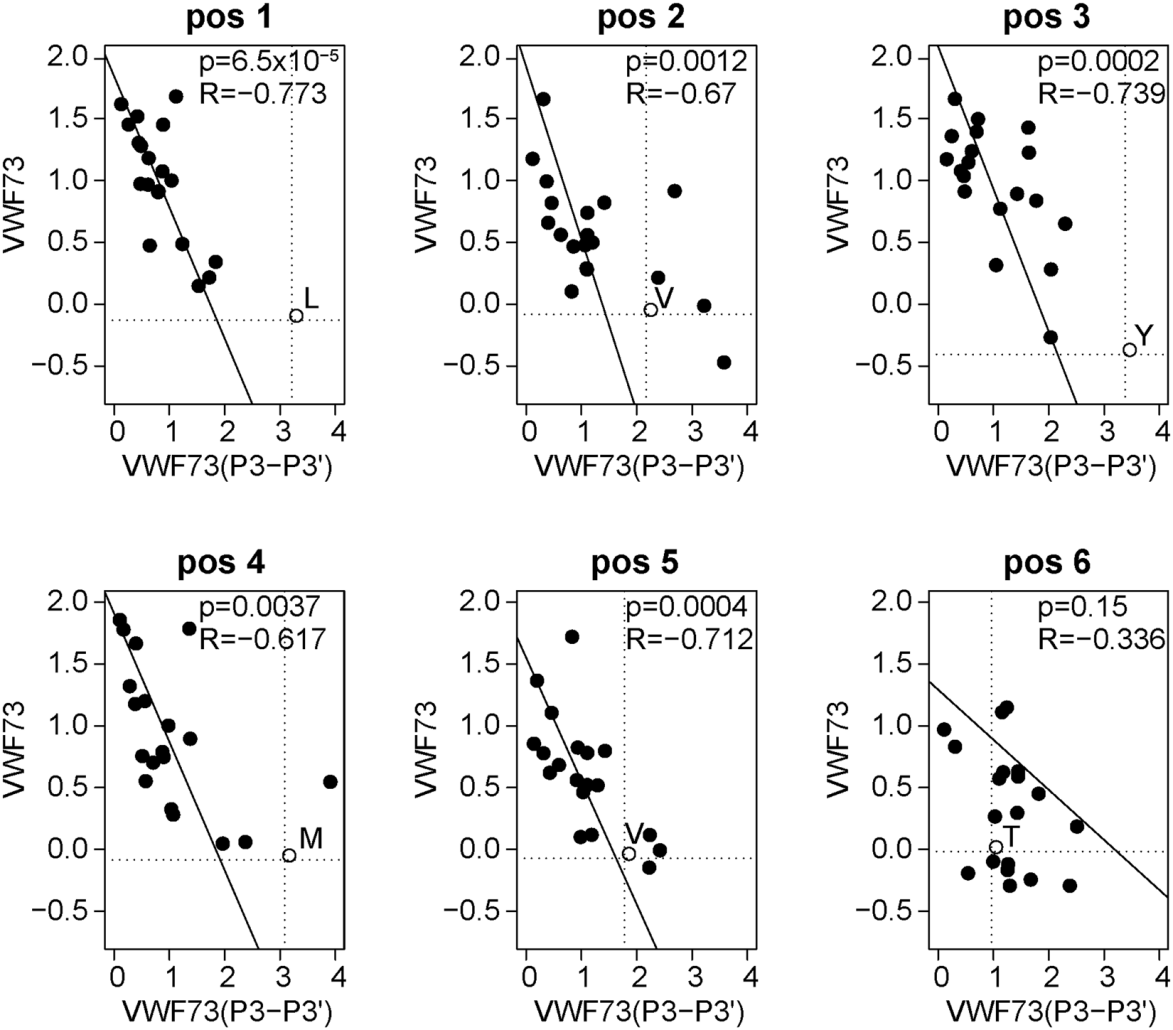
Correlation between ADAMTS13 selection of VWF73 mutagenesis and VWF73(P3-P3′) libraries. ADAMTS13 selection of the VWF73(P3-P3′) library was compared to previously reported VWF73 mutagenesis data^30^, where substitutions tended to inhibit cleavage of wild type VWF73. Thus, the enrichment for each substitution within the P3-P3′ interval^30^ was correlated to the relative proportions of each amino acid in enriched compared to depleted peptides from the VWF73(P3-P3′) library selection. Each panel represents an amino acid position, and each data point represents an amino acid substitution. The wild type amino acid at each position is indicated at the intersection of the dashed lines. Values represented in each plot correspond to p-values calculated for each correlation (top) and the R value (bottom) for the linear regression (line).

We recently reported a comprehensive kinetic characterization for nearly every amino acid substitution at each position of VWF73^30^. Comparing these previous results with the current analysis revealed a strong correlation between experimental datasets (Fig. 7). Variants at positions 1, 3, and 5 showed the strongest correlation (R > 0.7), whereas position 6 exhibited the weakest correlation (R of ~0.33). These data indicate that while most amino acid substitutions in the P3-P3′ interval inhibit proteolysis relative to wild type VWF^30^, many changes are tolerated and can ultimately be cleaved by ADAMTS13.

## Discussion

We have generated a comprehensive catalog of the substrate specificity for thrombin and ADAMTS13 based on a phage display library of random 6 amino acid peptides. As expected, thrombin exhibited strong preference for Arg and a weaker preference for Lys, consistent with the P1 requirements of known natural substrates. In addition to defining preferred amino acids, our data reveal negative regulators of thrombin substrate recognition including acidic amino acids, Pro at any position except P2, and Ser at any position except P1′. In contrast to thrombin, ADAMTS13 exhibited poor recognition of random hexamer substrates. The number of cleaved peptides by ADAMTS13 was expanded >17-fold when residues P3-P3′ of VWF73, a known ADAMTS13 substrate that also contains exosite binding residues, were replaced by random 6 amino acid peptides. These data suggest that exosite interactions are required for substrates to gain access to the ADAMTS13 active site. Overall, these data provide the most comprehensive set of substrate recognition peptides for these proteases, and illustrate distinct modes of specificity determination.

### Thrombin

Thrombin is the final effector protease of blood coagulation and participates in both amplification and attenuation of the clotting system. As such, thrombin is one of the most widely characterized proteases in the human genome^7,34–36^. Our data identify a comprehensive set of thrombin-recognized peptides, exceeding 6,700 unique peptide sequences, expanding on previous reports. Our findings demonstrate the most restricted amino acid diversity within the P2-P1′ interval, consistent with the idea that 3 amino acid peptide substrates can effectively discriminate thrombin specificity^34^. Although P2 Pro dominates thrombin natural substrates, it is not an absolute requirement for proteolysis at P1 Arg, with other low molecular weight amino acids such as Ala, Val, and Leu also found at P2 in our data. By contrast, bulky or electrostatic amino acids at this position abrogated substrate recognition. These observations are consistent with the crystal structure of thrombin, illustrating an apolar S2 pocket marked by Trp215 of thrombin^37,38^. The comparably shallow S1′ pocket^39^ also excludes bulky amino acids, consistent with the over-representation of lower molecular weight amino acids at the P1′ position. Bulky hydrophobic amino acids, such as Trp, Tyr, and Met, emerged in the extended substrate positions (P5-P3 and P2′-P4′) consistent with previous library screens and natural thrombin substrate alignments^7^. These amino acids are expected to fill vacant pockets previously observed in crystal structures of thrombin complexed with hirudin, and likely stabilize substrate interactions^39–41^.

These data are consistent with previous phage display screens of thrombin (summarized in^7^), but with a few notable exceptions. Although previous studies have demonstrated a preference for Gly at P2, our data show a much higher proportion of Gly in the uncleaved peptides, suggesting a net-antagonistic role in thrombin substrate recognition. This difference can likely be explained by the fact that Gly was the most abundant amino acid in our library. As a result Gly is expected to be found in cleavable peptides by chance and does not itself support thrombin interactions with substrates. Indeed, Gly was previously identified at all amino acid positions^7^, further supporting a nonspecific role in substrate recognition. These data also highlight the power of high throughput sequencing coupled to substrate phage display. The simultaneous quantifying of enrichment and depletion for millions of unique peptide sequences in a single protease reaction provides greater power to detect subtle effects on substrate recognition than was previously possible.

### ADAMTS13

VWF is currently the only known substrate for ADAMTS13^11^, which could suggest a narrow substrate profile. Consistent with this hypothesis, ADAMTS13 cleaved only 96 peptides from the random peptide library. The enriched peptides preferentially contained bulky hydrophobic amino acids but revealed no coherent motif, suggesting poor substrate recognition within this comprehensive library. These findings are consistent with a previous report demonstrating a greater than 1500-fold reduction in the k_cat_/K_M_ for proteolysis of VWF by ADAMTS13 in the absence of exosite interactions^17^. Our library theoretically surveys all possible 6 amino acid peptide sequences, and therefore confirms the notion that ADAMTS13 does not efficiently recognize short peptidyl substrates^17,21^.

Recently, a mechanism of ADAMTS13 auto-regulation was described in which COOH-terminal CUB domains interacting with the NH_3_-terminal spacer domain^42,43^. A mechanism was proposed whereby exosite engagement activates ADAMTS13 by relieving this auto-regulation in addition to aligning the substrate scissile bonds toward the active site. Consistent with this mechanism, we observed that ADAMTS13 recognition of a random peptide library was expanded >17-fold when the random peptide library was expressed within the context of an exosite-binding substrate. These data may suggest that access to the active site is impaired when ADAMTS13 adopts its closed conformation.

Alignment of cleaved peptides revealed a distinct substrate recognition motif for ADAMTS13. Our data indicate that long-chain aliphatic amino acids at P3 (including Leu, Ile, and Met) are a dominant feature for ADAMTS13 substrate recognition, consistent with previous findings which highlight the importance of the P3 residue for ADAMTS13 substrate recognition^44^. Overall, substrate recognition for ADAMTS13 exhibits a general requirement for aliphatic and aromatic residues throughout, including Tyr at P1, and Leu, Tyr, Met, and Phe at P1′. Although no crystal structure of the ADAMTS13 metalloprotease domain is currently available, the structure for the corresponding domain in ADAMTS5 (which shares 28% amino acid sequence identity and 42% similarity with ADAMTS13) has been solved^45^. This structure reveals a hydrophobic active site cleft with a deep S1′ pocket, characteristic of other metalloproteases of the metzincin family, that is known to accept bulky aliphatic residues at the P1′ position of substrates. However, the structure of the ADAMTS5 protease domain does not identify a binding site for the P3 residue^30,44^. Previous studies demonstrated that ADAMTS13 residues Asp187-Arg193 forms a subsite within the metalloprotease domain that flanks the active site and contributes to recognition of the VWF scissile bond^46^. Interestingly, the charged residues within this loop (D187, R190, and R193) appeared to make the greatest contribution to substrate recognition. How these residues influence the selectivity of peptides containing bulky hydrophobic amino acids in the VWF73(P3-P3′) library remains to be determined. Overall, these data suggest that ADAMTS13 is capable of recognizing and cleaving proteins other than VWF only if exosites are simultaneously engaged. The consensus motif and list of cleavable peptides may facilitate the discovery of novel physiological substrates of ADAMTS13.

We previously interrogated the interaction between ADAMTS13 and VWF73 using a comprehensive mutagenesis substrate phage display library and showed that the P3-P2′ interval is among the most critical regions driving ADAMTS13 substrate recognition^30^. The data reported here are highly concordant with this previous report, providing a more detailed investigation of the P3-P3′ interval. Together, these studies provide a broad framework for comprehensive protease profiling that complement or expand upon existing technologies^3,10,47,48^.

However, we acknowledge a number of potential limitations to our approach. First, this technique does not define the P1-P1′ site of cleavage for each peptide identified. In the case of thrombin, the strategy of aligning peptides by fixing an Arg residue is supported by extensive investigation over many decades, as well as the identification of very similar motifs for peptides containing a single Arg compared to peptides containing multiple Arg residues. For ADAMTS13 cleavage of VWF73(P3-P3′), exosite interactions within VWF73 may restrict ADAMTS13 cleavage to the 3^rd^ or 4^th^ position of the hexamer library, though cleavage elsewhere in the P3-P3′ interval cannot be excluded. For example, the presence of Tyr and Phe at position 4 of cleaved peptides may be indicative of the P1 residue shifting from position 3 in certain peptides. As a result, the motif generated from the VWF73(P3-P3′) library may be incomplete.

The reaction conditions employed here are expected to result in the proteolytic reaction proceeding to completion, providing great sensitivity to detect even weak substrates, but limiting quantitative comparison among cleaved peptides. For example, 5 of the most significantly cleaved peptides from the VWF73(P3-P3′) library (Supplementary data 2) did not cleave as efficiently as wild type VWF73, which was 135^th^ most heavily selected by the cleavage assay (Table 2). Thus, the possibility that select peptides within in this library may still exhibit increased efficiency as ADAMTS13 substrates compared to WT cannot be excluded.

Despite these limitations, our findings demonstrate the power of coupling substrate phage display to high throughput sequencing to provide a rapid and robust platform for comprehensive protease profiling. Current high throughput sequencing technology provides the capacity to sequence ~300 million molecules in parallel (Illumina). This capacity allows precise enrichments to be calculated for every library clone, and statistical interpretations of the data after a single round of selection. This approach avoids biases in phage infection and re-amplification that commonly confound traditional phage display biopanning experiments^49^. Furthermore, recent advances in oligonucleotide array synthesis allow for rationally designed substrate libraries and more precise control over library composition^50,51^. As these technologies continue to improve, the capacity to investigate more comprehensive libraries will expand and yield new insights into protease specificity determination. Ultimately, these studies could facilitate the identification of novel physiological protease substrates, development of more specific biochemical or clinical tools to assess protease activity, and support the development of specific protease inhibitors to treat important human diseases.

## Methods

### Phagemid Modification

The fUSE55 vector^52^ was modified to contain a cotranslational-translocation signaling sequence and NH_2_- and COOH-terminal epitope tags (See Table S1 for complete oligonucleotide list). A FLAG tag was first inserted into the phagemid, pAY-E^53^, at the NotI and SgrAI sites using annealed oligomers, **P1** and **P2**, generating pAY-FE. Tandem FLAG and E epitope tags followed by a glycine-serine rich linker were amplified from pAY-FE with primers, **P3** and **P4**, and inserted into fUSE55 at the BglI site, generating fUSE65. The TorT (i.e., cotranslational-translocation) signaling sequence was fused to transcriptional regulatory elements of fUSE55 by PCR using primers **P5-P7**, and subsequently inserted at the BsrGI and SfiI sites of fUSE65 to generate fUSE66. For fUSE67, oligomers **P8** and **P9** were annealed and extended using standard PCR protocols and inserted into fUSE66 at the SfII and SgrAI sites. The resulting features of fUSE67 vector are arranged: 5′-TorT signaling sequence, FLAG tag, T7 tag, multiple cloning site, E tag, glycine-serine rich linker, and gIII-3′. All expected modifications were verified by Sanger DNA sequencing. All oligonucleotides were from Integrated DNA Technologies (Coralville, Iowa).

### Construction of substrate phage display libraries

Three distinct phage display libraries were generated to evaluate the substrate recognition patterns of thrombin and ADAMTS13. The random nucleotide libraries were either inserted into FUSE67, or designed to contain a FLAG-tag 5′ to the variable region before cloning into the FUSE55 phage display vector^52,54^. Both FUSE67 and FUSE55 place the substrate on all copies of the PIII protein of M13 filamentous phage.

To construct the random 6 amino acid substrate phage display library, the NNK degenerate codon series was used, where N represents an equal 25% proportion of A, C, G, and T, and K represents equal 50% proportion of G and T. Thus, 10 ng of the NNK oligonucleotide **L1** was used as a template in a PCR reaction containing 1 μM **S1** and 1 μM **AS1** primers (Table S2) using the following thermal profile for 30 cycles: 95 °C (30 s), 60 (30 s), 72 (30 s).

The PCR product was gel purified on 1.5% agarose and extracted using the QIAquick Gel Purification Kit (Qiagen), and digested with Bgl1 (NEB). All restriction digested products were prepared for ligation using agarose gel purification followed by electroelution using the ELUTRAP system (GE Healthcare). The digested and purified oligonucleotides were ligated into 1 μg of FUSE55 using a 6:1 molar ratio (insert:vector). The ligation mixture was incubated at 16 °C overnight, precipitated, and resuspended in TE buffer (20 mM TRIS-HCl, pH 8.0, 1 mM EDTA). The ligation product was electroporated into MegaX DH10B *E. coli* (Invitrogen), and the library was titrated, revealing a total library depth of 2.5 × 10^8^ independent clones.

Random 6 amino acid peptide libraries were also constructed in the context of VWF73 (Asp1596-Arg1668 of VWF), replacing the codons for Leu8-Thr13 with the degenerate codon series, NNK. Two approaches for the library construction were undertaken. In the first approach (VWF73(P3-P3′)-1), the NNK randomization was tailed onto the forward primer with 1 ng of VWF cDNA in pBlueScript SK+ used as template in a PCR reaction containing 1 μM **S2** and 1 μM **AS2** (Table S4), using Herculase II (Agilent). The PCR product was gel purified as above and used as template in a PCR reaction containing 1 μM **S3** and 1 μM **AS2**. The PCR product was gel purified as above and used as template in a final PCR reaction containing 1 μM **S4** and 1 μM **AS2**. The PCR product was gel purified as above. In all cases, the PCR thermal profile was: 95 °C (30 s), 62 (30 s), 72 (30 s), repeated for 20 cycles. A second library was constructed (VWF73(P3-P3′)-2, where the randomized oligonucleotide was used as a template to account for possible nucleotide bias in VWF73(P3-P3′)A. A single PCR reaction was assembled containing 1 nM **L2**, 1 nM **AS3**, 1 nM **AS4**, 1 μM **AS5**, and 1 μM **S5** (Table S5) using Herculase II. The PCR thermal profile was: 95 °C (30 s), 60 (30 s), 72 (30 s), repeated for 30 cycles.

In all approaches, the PCR products were digested with either Bgl1 or Asc1 and Not1, gel purified using ELUTRAP, then ligated into 1 μg FUSE55 or FUSE67 at a 6:1 molar ratio (insert:vector) overnight at 16 °C. The ligation product was precipitated, resuspended in TE buffer, and electroporated into MegaX DH10B *E. coli*. The libraries were titrated onto 30 μg/mL tetracycline Luria Broth (LB) agar plates revealing 3 × 10^7^ independent clones for VWF73(P3-P3′)A and 1 × 10^7^ independent clones for VWF73(P3-P3′)B. For the two VWF73(P3-P3′) libraries, no major differences in library composition were detected by high throughput sequencing, and datasets were combined for final analysis.

### Panning

The phage libraries were prepared as previously described^30^. Approximately 1 × 10^10^ phage were added to 1 mL TBS-B (20 mM Tris-HCl pH 7.4, 150 mM NaCl, 1% BSA) containing 50 μL anti-FLAG agarose beads (Sigma), and mixed at room temperature for 2 hr. The beads were recovered by gentle centrifugation (3000 × g for 1 min) and washed 5 times with TBS-B. The phage-coated beads were then resuspended with 500 μL reaction buffer (20 mM Tris-HCl, pH 7.4, 150 mM NaCl, 5 mM CaCl_2_, 10 μM ZnCl_2_, and 1% BSA) containing 5 nM thrombin (Hematologic Technologies) or 5 nM ADAMTS13 (R&D Systems). These reaction conditions have previously been shown to result in efficient hydrolysis of peptidyl substrates for both thrombin^55^ and ADAMTS13^56^. The reaction was incubated overnight with end-over-end mixing at room temperature. The beads were recovered by centrifugation, and the supernatant containing phage displaying cleaved peptides was recovered. For the control samples containing no protease, unreacted phage bound to anti-FLAG beads were eluted using 500 μL 0.15 mg/ml 3X FLAG peptide. Single stranded DNA (ssDNA) was prepared as previously described^30^.

### Deep sequencing

Unselected and selected phage ssDNA were used as templates in PCR reactions to prepare samples for high throughput sequencing to evaluate enrichment following panning, as previously described^30^. For all samples, an initial barcoding PCR was performed using primers listed in Table S3 for the random peptide substrate phage display library and Table S6 for VWF73(P3-P3′). The thermal profile was: 98 °C (30 s), 62 °C (30 s), 72 °C (30 s). The number of cycles was determined empirically to prevent product laddering, assessed by agarose gel electrophoresis. To complete the assembly of Illumina library adapters, a second PCR was performed using 10 ng of the barcoded PCR product as template and 0.5 μM of **PE1seq** and **PE2seq** primers (Table S3). The thermal profile was: 98 °C (30 s), 60 °C (30 s), and 72 °C (30 s). PCR products were gel purified on 1% agarose.

Illumina library quality was assessed by qPCR using the Library Quantification Kit (KK4835, Kapa Biosystems) and the Agilent DNA 1000 Bioanalyzer kit (5067-1504, Agilent), according to manufacturer’s instructions. Libraries were sequenced on a HiSeq2500 (Illumina) using paired-end 50 base pair reads in Rapid Mode.

### Recombinant phage and peptide validation

The results of the VWF73(P3-P3′) screen were validated in part using purified recombinant peptide clones. Recombinant phage and peptides were purified and k_cat_/K_M_ values determined as previously described^53^. All oligonucleotides used to assemble the clones are provided in Table S8.

### Sequencing analysis pipeline and QC analysis

Sequence filtering and peptide analysis were performed using an in-house pipeline written in Python and are available for download (github.com/tombergk/NNK_VWF73/). A number of quality filters were applied to the paired-end reads from the.fastq files (Figure S1). First, one of the reads from each pair (forward or reverse) was compared to one of three 8 bp seed sequences within the forward primer region to orient the sequence. The multiple seeds allowed for sequencing errors to be tolerated at this initial stage without discarding the read. Second, a perfect match of nucleotides between the sense and antisense reads was required within the variable coding region. This highly stringent quality filter should reduce sequencing errors within the library to 0.01%, assuming a 1% error rate per sequence^57^. Finally, a base pair quality score of at least 5 out of 40 was required from each position within variable coding region. Stop codons were evaluated (see Results) but removed from subsequent analyses. Because the FUSE55 (and FUSE67) phage display system places a displayed peptide on all PIII proteins, a stop codon within the library should abrogate PIII production and prevent phage assembly. As a result, any occurrence of stop codons in the library is likely due to sequencing errors, although occasional ribosome read-through cannot be excluded. All paired-end sequences that passed the above quality filters were translated into corresponding peptides and the occurrence of each unique peptide was recorded. Biases in amino acid content between the random 6 amino acid peptide library and VWF73(P3-P3′) are shown in Table S7.

Generated.fastq files have been deposited to the NCBI Sequence Read Archive (project accession number #PRJNA356764) found at https://www.ncbi.nlm.gov/sra. The project encompasses 3 sets of paired-end high throughput sequencing.fastq files used in our pipelines: #SRR5097080, #SRR5097081, #SRR5097082.

### Motif definition and determination

Peptides containing a minimum of 4 reads combined in selected and unselected controls were analyzed. Enrichment and depletion of peptides was assessed using the DESEQ. 2 software package^58^, which estimated variance-mean dependence in peptide counts from selected and unselected phage and tested for differential expression using a negative binomial distribution. Peptides with Benjamini-Hochberg^59^ adjusted p-values (p_FDR_) < 0.05 were considered significant for both enrichment and depletion. All significantly enriched and depleted peptides from the selections are available as supplemental files. Amino acid frequency plots and heatmaps were created using the iceLogo package^60^, where the ratio of amino acid frequencies in the enriched peptides was compared to depleted peptides. In the case of thrombin, all peptides containing a single Arg were aligned and centered around Arg to assess the amino acid dependency in this context.

## Electronic supplementary material

Supplementary Figures and Tables

Supplementary Data 1

Supplementary Data 2
