# Supplementary material for: High throughput protease profiling comprehensively defines active site specificity for thrombin and ADAMTS13
Source: Sci Rep. 2018 Feb 12;8:2788. doi: 10.1038/s41598-018-21021-9 (PMC5809430; doi:10.1038/s41598-018-21021-9)
Supplement: Supplementary file 1 — Supplementary Figures and Tables [file 41598_2018_21021_MOESM1_ESM.pdf]

Supplement

High throughput protease profiling comprehensively defines active site specificity for thrombin and ADAMTS13

Colin A. Kretz[1*] and Kärt Tomberg[3*], Alexander Van Esbroeck[4], Andrew Yee[2], and David Ginsburg[2,3,5]

[1]Department of Medicine, McMaster University and the Thrombosis and Atherosclerosis Research Institute, Hamilton, Ontario, Canada, [2]Life Sciences Institute, University of Michigan, [3]Department of Human Genetics, University of Michigan, [4]Department of Electrical Engineering and Computer Science, [5]Howard Hughes Medical Institute and Departments of Internal Medicine and Pediatrics, University of Michigan, Ann Arbor, MI. *These authors contributed equally to this work.

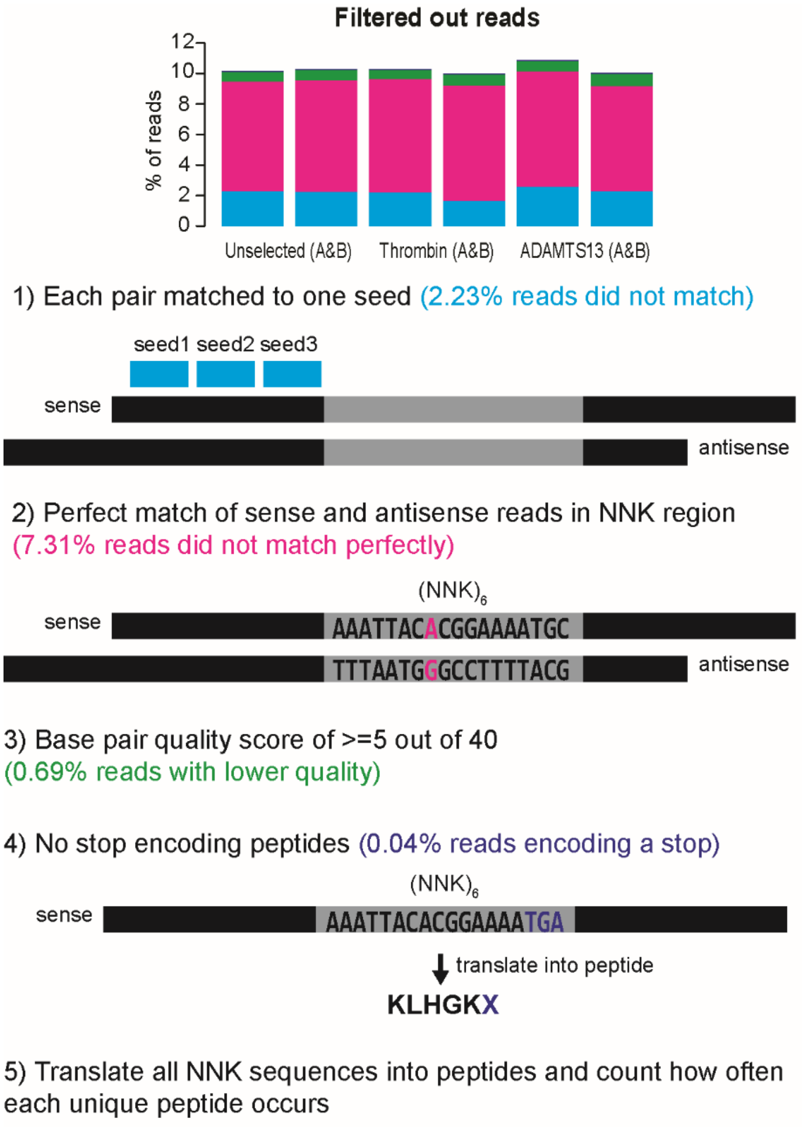

Figure S1: Data Analysis Pipeline

Approximately 10% of the paired-end reads were removed from the dataset by four different quality filters. Removed reads included 1) reads that did not match any of the given seed sequences to orient the sequence (light blue), 2) did not have perfect match between sense and antisense reads within the NNK region (magenta), 3) had quality score <=5 out for 40 at any NNK position (green), 4) encoded for a stop codon (dark blue, not visible).

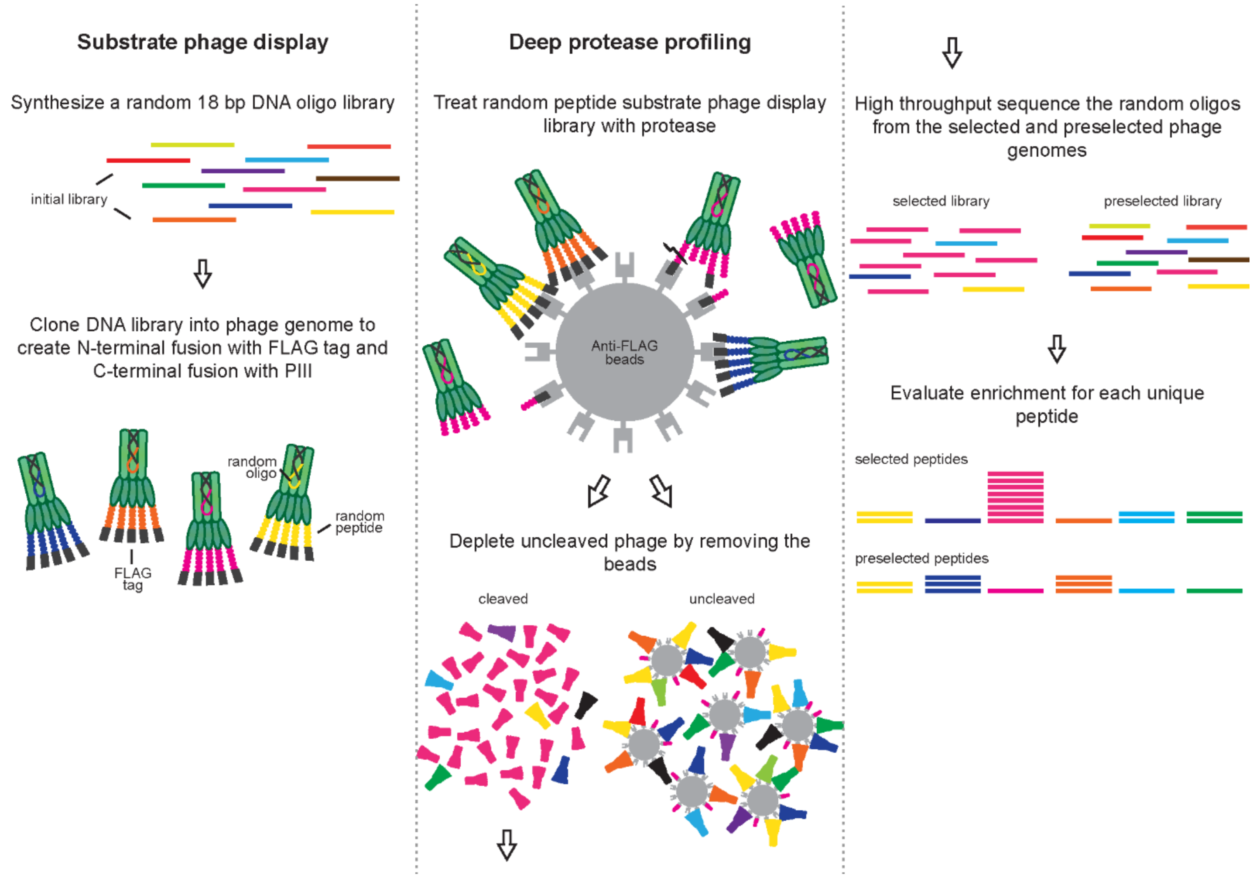

Figure S2: Deep protease profiling

(A) Substrate phage display employs a random peptide library cloned between an epitope tag and the phage PIII protein, which is anchored to the phage body. (B) Initially, phages displaying a recombinant peptide are captured using anti-FLAG agarose beads. Following incubation with the protease, cleaved phages are released from the beads and separated from remaining uncleaved phage. (C) Single stranded phage DNA from the cleaved phage pool is prepared and the library inserts are amplified by PCR and appropriate adapters are added for high throughput sequencing. Enrichment relative to the unselected library is evaluated by counting the occurrence of each unique peptide in the sequencing data.

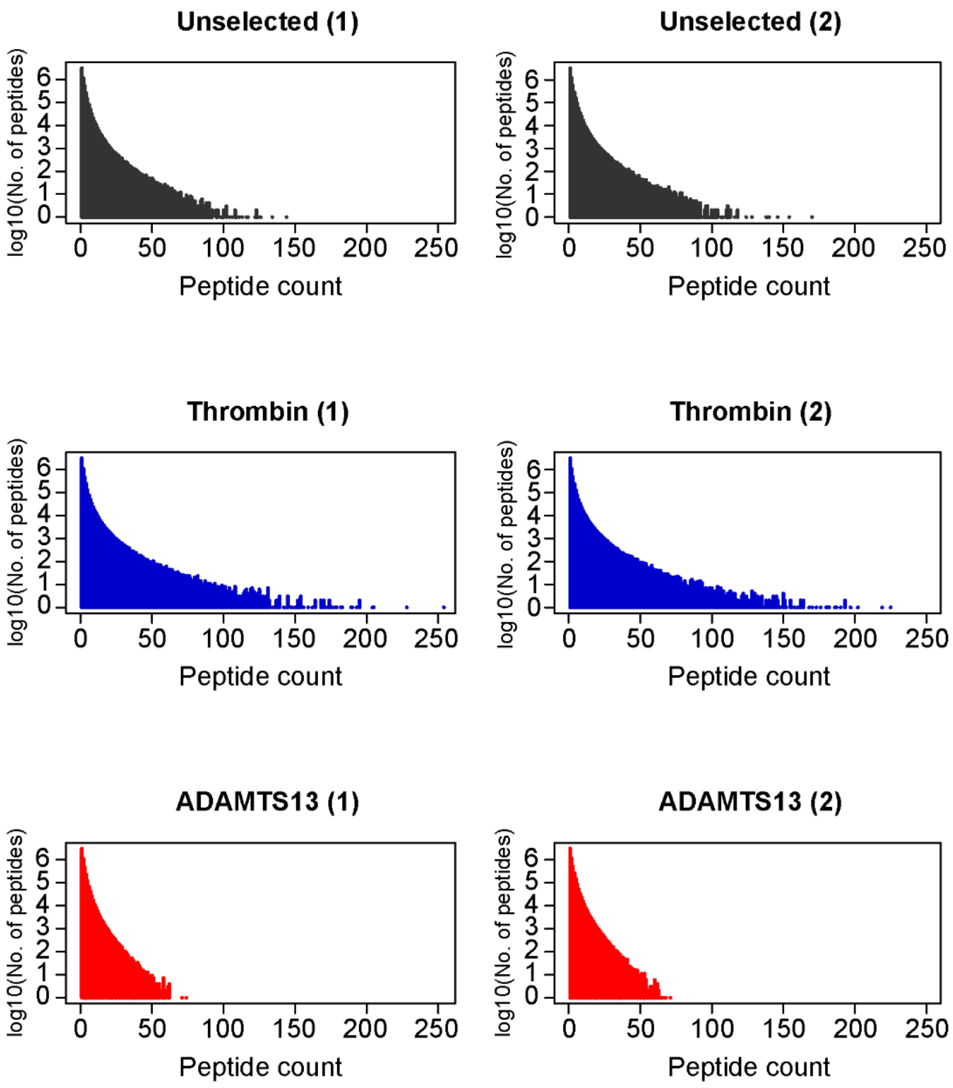

Figure S3: Read distribution in random peptide library
Histograms representing unique peptide counts in the random peptide for unselected (black), thrombin selected (blue), and ADAMTS13 selected (red) phage populations. Most peptides are seen only once or twice in all treatments. For final analysis, we required a minimum of 4 sequencing reads per peptide.

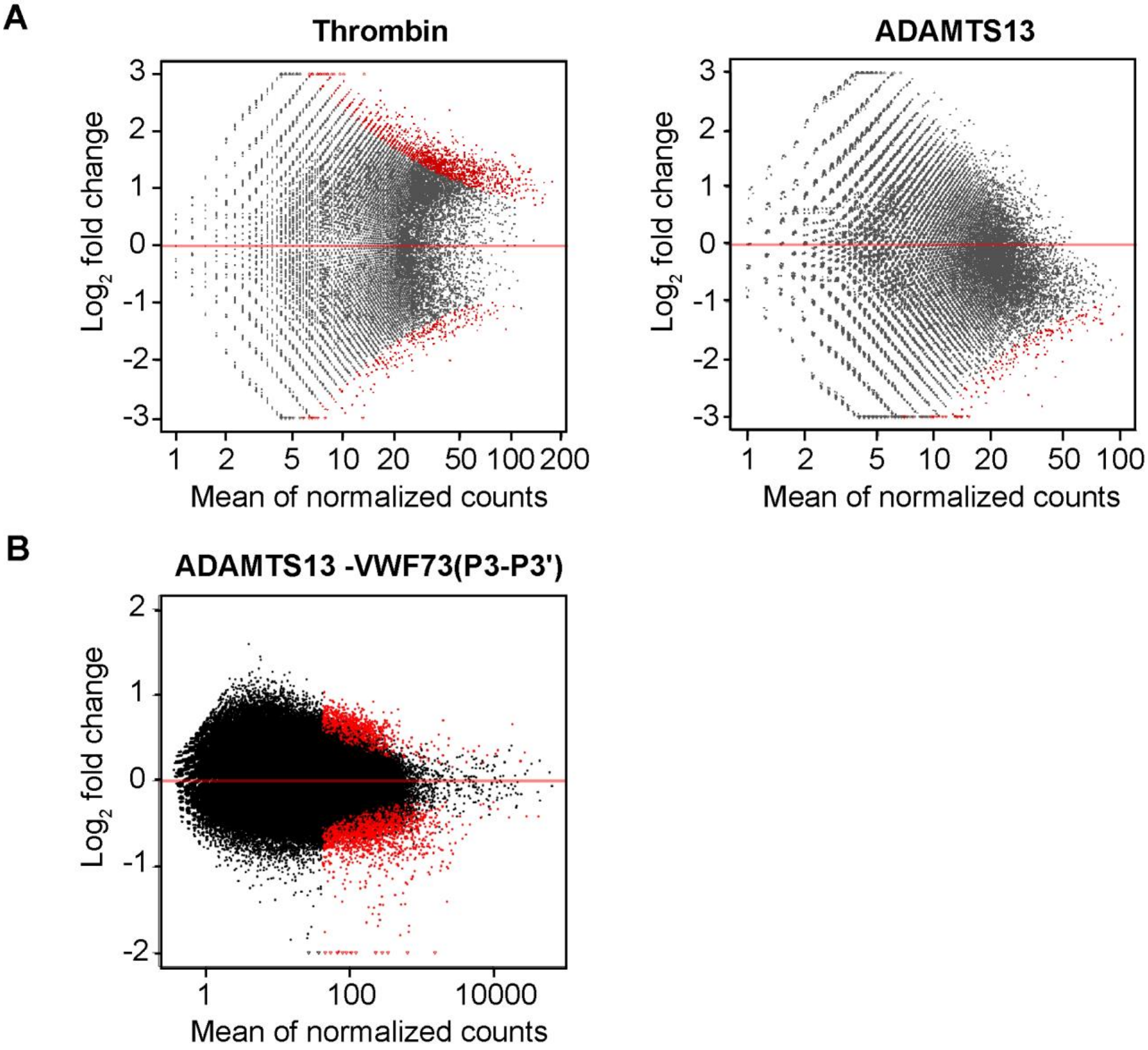

Figure S4: Deseq2 enrichment plots

The MA-plot derived from Deseq2 sofware (49) illustrates the log2 fold changes attributable to a given unique peptide over the mean of normalized counts. Points which fall out of the window are plotted as open triangles pointing either up or down. This figure illustrates the statistical treatments of the enrichment data that are required to control for stochastic variation in read count. Only those data points with $p_{adj} < 0.05$ (red) are used in subsequent analyses. A, the MA plots for thrombin or ADAMTS13 selection of the random 6 amino acid peptide library. B, the MA plot for ADAMTS13 selection of the VWF73(P3-P3') library.

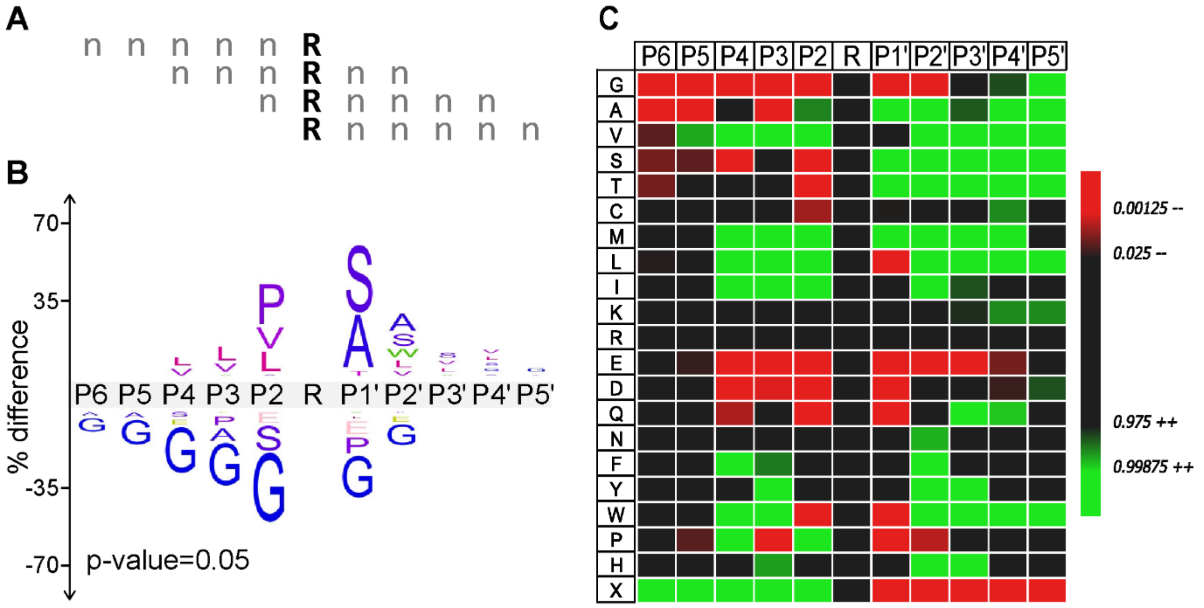

Figure S5: Thrombin motif of peptides containing a single Arg
An iceLogo plot of peptides significantly enriched (3152 peptides) vs depleted (906 peptides) after selection by thrombin was created. (B) The plot shows the frequency of each amino acid per position in cleaved (top) and uncleaved (bottom). Amino acids are colored by biochemical property. (C) A heatmap shows a different representation of the same data, and more clearly indicates the performance of each amino acid at each position. Green shows amino acids that potentiate cleavage by thrombin, and red shows amino acids that antagonize cleavage by thrombin.

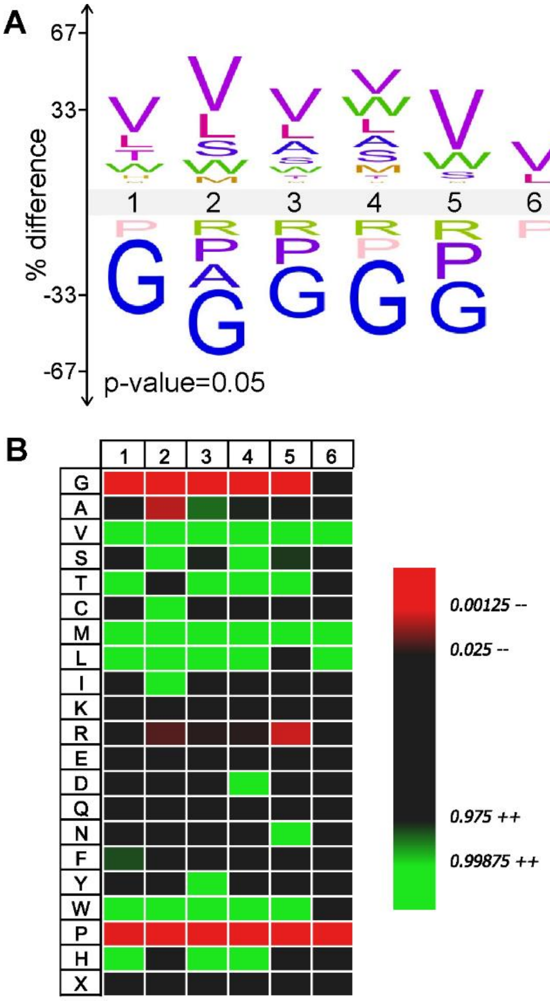

Figure S6: Amino Acid motif for ADAMTS13 enrichment and depletion
Frequency plot of all significantly enriched and depleted peptides after selection by ADAMTS13 using iceLogo as in Figure S5.

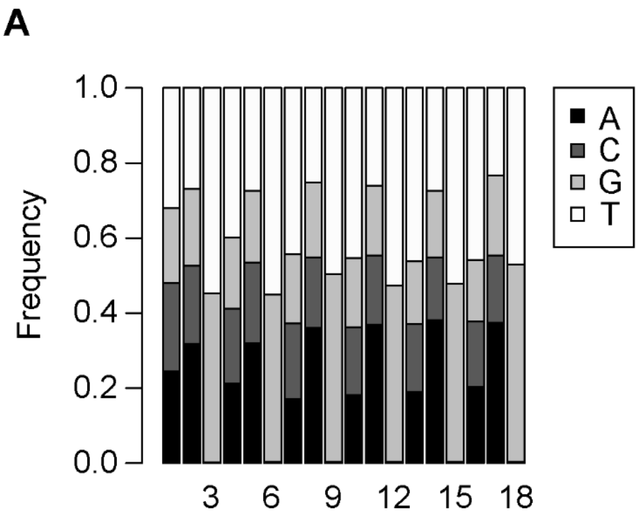

Figure S7: Nucleotide distribution in VWF73(P3-P3') libraries
The frequency of each nucleotide at the 18 position of VWF73(P3-P3') library A and B is shown, confirming NNK randomization.

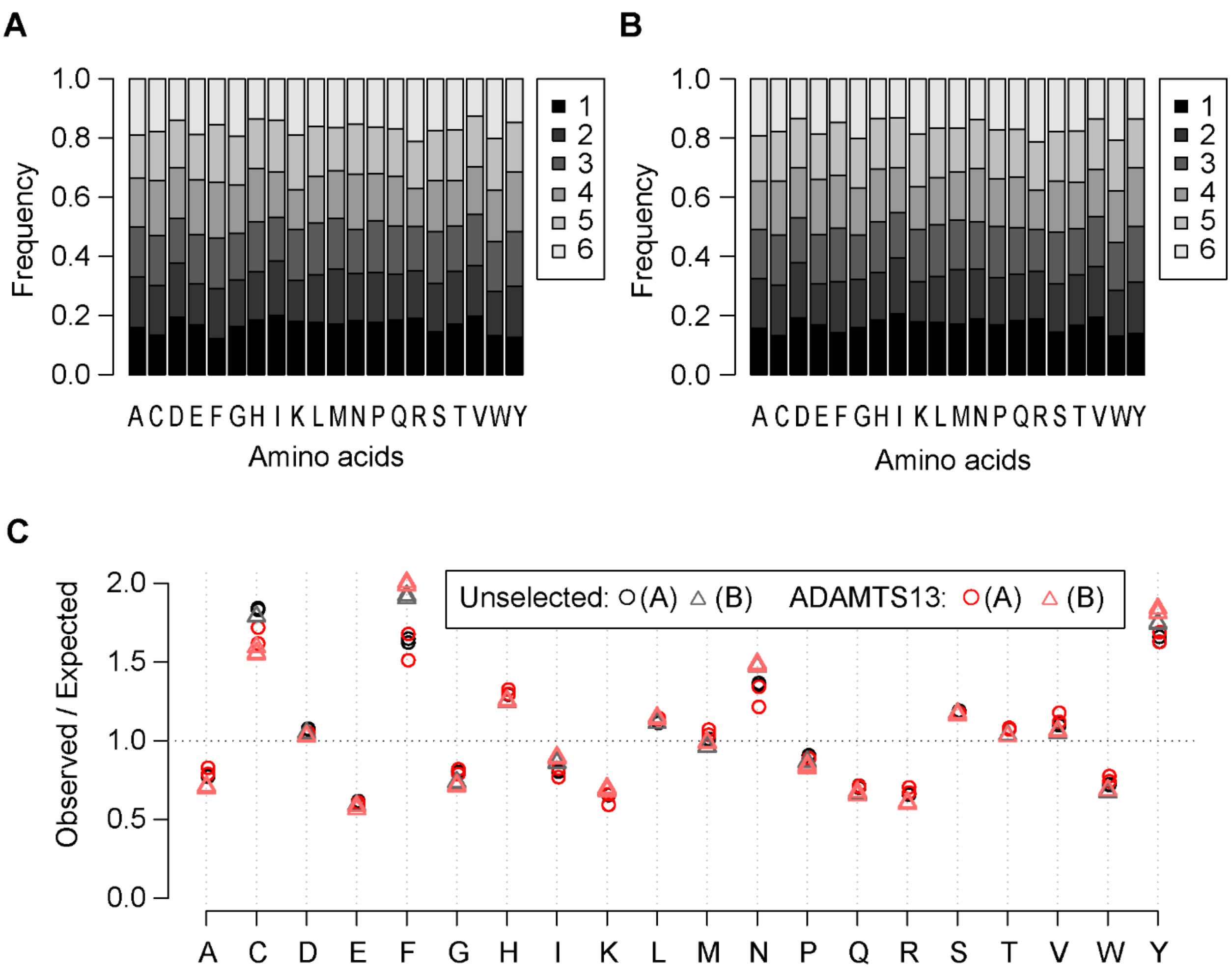

Figure S8: Amino Acid distribution in VWF73(P3-P3') library
A and B) The proportion of each amino acid at the 6 positions of the VWF73(P3-P3') library A and B, respectively. C) The frequency of each amino acid in the unselected libraries and ADAMTS13 selected library was compared to the codon frequencies within the NNK randomization scheme. These data show differences in amino acid frequency compared to expected, that are different than in the original random peptide library (Figure 1C), implying a role of the VWF73 peptide sequence in library bias.

Supplementary Tables

Table S1: Primer sequences for FUSE67 modifications from FUSE55 parent vector

Primers/ Oligomer	Sequence
P1	/5'Phos/GGCCGCAGGTGGTGGTGACTACAAGGACGATGACGATAAAGGAGGAGGAGGTGCG
P2	/5'Phos/CCGGCGCACCTCCTCCTCCTTTATCGTCATCGTCCTTGTAGTCACCACCACCTGC
P3	TCGGCCGACGTGGCCCCGGCGCGCCCTGCAGGTCGACGCGGCCGCAGGTGGTGGTGACTA
P4	TCGGCCCCAGAGGCTTCACCGGAACCGGAGCCGCCACCGGAA
P5	CGCCTGGTCTGTACACCGTGCATCTGT
P6	AGCAGCAGGAACAGCAGAACACGCATGTTGAAAATCTCCAAAAAAAAAGGCTCCAAAAGG
P7	GGGGCCACGTCGGCCGAGAAAGCCGGCAGCATGAACAGAGACAGCAGCAGGAACAGCAGA
P8	TCGGCCGACGTGGCCGACTACAAGGACGATGACGATAAAGGTATGGCATCAATGACAGGAGGACAACAAATGGGTGGTGGTTCGGGCGCG
P9	CGGCACCGGCGCACCGGAACCACCACCTGCGGCCGCGTCGACCTGCAGGGCGCGCCCGAACCACCACCCATTTGTTGTCCTCCTGTCATT

Table S2: Random 6 amino acid peptide library preparation oligonucleotides

**Name**	**Sequence (5-3)**
L1	TCGGCCGACGTGGCCGACTACAAGGACGATGACGATAAAGGAATGGCATCAATGACAGGAGGACAACAAATG**(NNK)$_6$**ATGGCCTCTGGGGCCGAAACT
S1	TCGGCCGACGTGGCCGACTAC
AS1	AGTTTCGGCCCCAGAGGCCA

Table S3: $(NNK)_6$ Deep sequencing library preparation primers

Primers used for the preparation of Illumina sequencing libraries from phage ssDNA template are shown. Primers include Illumina primer hybridization domain (bold), 6N wobble for improved cluster diversity, unique barcodes for both forward and reverse primers (lowercase), and a FUSE55 vector hybridization domain. PCR products from barcoding primers were isolated and used as a template in a PCR reaction that completed the Illumina adapter sequence using PE1-seq and PE2-seq.

Name	Sequence (5'-3')
NGSa-S1	**ACACTCTTTCCCTACACGACGCTCTTCCGATCT**NNNNNNgaatc ACAGGAGGACAACAAATG
NGSa-AS1	**CTCGGCATTCCTGCTGAACCGCTCTTCCGATCT**NNNNNNgtcta ACTTTCAACAGTTTCGGCCC
NGSb-S1	**ACACTCTTTCCCTACACGACGCTCTTCCGATCT**NNNNNNtctga ACAGGAGGACAACAAATG
NGSb-AS1	**CTCGGCATTCCTGCTGAACCGCTCTTCCGATCT**NNNNNNcagag ACTTTCAACAGTTTCGGCCC
NGSc-S1	**ACACTCTTTCCCTACACGACGCTCTTCCGATCT**NNNNNNcagtc ACAGGAGGACAACAAATG
NGSc-AS1	**CTCGGCATTCCTGCTGAACCGCTCTTCCGATCT**NNNNNNttcac ACTTTCAACAGTTTCGGCCC
NGSd-S1	**ACACTCTTTCCCTACACGACGCTCTTCCGATCT**NNNNNNagtct ACAGGAGGACAACAAATG
NGSd-AS1	**CTCGGCATTCCTGCTGAACCGCTCTTCCGATCT**NNNNNNagtct ACTTTCAACAGTTTCGGCCC
NGSe-S1	**ACACTCTTTCCCTACACGACGCTCTTCCGATCT**NNNNNNtgcaa TCAATGACAGGAGGACAACAAATG
NGSe-AS1	**CTCGGCATTCCTGCTGAACCGCTCTTCCGATCT**NNNNNNtcagg ACTTTCAACAGTTTCGGCCC
NGSf-S1	**ACACTCTTTCCCTACACGACGCTCTTCCGATCT**NNNNNNacagt TCAATGACAGGAGGACAACAAATG
NGSf-AS1	**CTCGGCATTCCTGCTGAACCGCTCTTCCGATCT**NNNNNNatgtc ACTTTCAACAGTTTCGGCCC
PE1seq	CAAGCAGAAGACGGCATACGAGATCGGTCTCGGCATTCCTGCTG AACCGCTCTTCCGATCT
PE2seq	AATGATACGGCGACCACCGAGATCTACACTCTTTCCCTACACGA CGCTCTTCCGATCT

Table S4: VWF73(NNK)$_6$-A Library Preparation Oligonucleotides

The following primers were used in a PCR reaction using VWF cDNA as a template. One nM of VWF73-S2 and VWF73-S3, 1 uM VWF73-S1 and VWF73-AS1, and 1 ng template. The resulting product was purified and digested with Bgl1 prior to cloning into FUSE55.

Name	Sequence (5'-3')
S2	CACTCGGCCGACGTGGCCGACTACAAGGACGATGACGATAAAG GAATGGCATCAATGACA
S3	AAAGGAATGGCATCAATGACAGGAGGACAACAAATGGACCGG GAGCAGGCGCCCAAC
S4	CGGGAGCAGGCGCCCAAC(NNK)$_6$GGAAATCCTGCCTCTGATGAG
AS2	TTCAACAGTTTCGGCCCCAGAGGCCATCCTCTGCAGCACCAGGT CAGG

Table S5: VWF73(NNK)$_6$-B Library Preparation Oligonucleotides

The following primers were used to generate a second VWF73(NNK)6 library from a synthetic template. The PCR was conducted with 1 uM VWF73-NNK S1 and VWF73-NNK AS3, 1 nM VWF73-NNK-AS1 and VWF73-NNK-AS2, and 1 ng VWF73-NNK-templ.

Name	Sequence (5'-3')
S5	TGGGTGGTGGTTCGGGCGCGCCCGGTGGTGGTTCGGACCGGGA GCAGGCGCCCAAC
L2	CGGGAGCAGGCGCCCAAC(NNK)$_6$GGAAATCCTGCCTCTGATGAG ATCAAGAGGCTGCCTGGAGACATCCAGGTGGTGCCC
AS3	CTGGATGAGGATAGGGGCATTGGGCCAGCCAATCCTCTCCAGCT CCTGCACGTTGGCATTAGGGCCCACTCCAATGGGCACCACCTGG ATGTC
AS4	CACCACCTGCGGCCGCGTCGACCTGCACCTCTGCAGCACCAGGT CAGGAGCCTCTCGGGGGAGCGTCTCAAAGTCCTGGATGAGGAT AGGGGCATT
AS5	CCGGCGCACCGGAACCACCACCTGCGGCCG

Table S6: VWF73(NNK)$_6$ Deep sequencing library preparation primers

Primers used for the preparation of Illumina sequencing libraries from phage ssDNA template are shown. Primers include Illumina primer hybridization domain (bold), 6N wobble for improved cluster diversity, unique barcodes for both forward and reverse primers (lowercase), and a VWF73 hybridization domain. PCR products from barcoding primers were isolated and used as a template in a PCR reaction that completed the Illumina adapter sequence using PE1-seq and PE2-seq (Table S2).

Name	Sequence
NGSg-S1	**ACACTCTTTCCCTACACGACGCTCTTCCGATCT**NNNNNNgaatcGAGCAGGCGCCCAAC
NGSg-AS1	**CTCGGCATTCCTGCTGAACCGCTCTTCCGATCT**NNNNNNgtctaATCAGAGGCAGGATTTCC
NGSh-S1	**ACACTCTTTCCCTACACGACGCTCTTCCGATCT**NNNNNNtctgaGAGCAGGCGCCCAAC
NGSh-AS1	**CTCGGCATTCCTGCTGAACCGCTCTTCCGATCT**NNNNNNcagagATCAGAGGCAGGATTTCC
NGSi-S1	**ACACTCTTTCCCTACACGACGCTCTTCCGATCT**NNNNNNcagtcGAGCAGGCGCCCAAC
NGSi-AS1	**CTCGGCATTCCTGCTGAACCGCTCTTCCGATCT**NNNNNNttcacATCAGAGGCAGGATTTCC
NGSj-S1	**ACACTCTTTCCCTACACGACGCTCTTCCGATCT**NNNNNNagtctGAGCAGGCGCCCAAC
NGSj-AS1	**CTCGGCATTCCTGCTGAACCGCTCTTCCGATCT**NNNNNNagtctATCAGAGGCAGGATTTCC
NGSk-S1	**ACACTCTTTCCCTACACGACGCTCTTCCGATCT**NNNNNNtgcaaGAGCAGGCGCCCAAC
NGSk-AS1	**CTCGGCATTCCTGCTGAACCGCTCTTCCGATCT**NNNNNNtcaggATCAGAGGCAGGATTTCC
NGSl-S1	**ACACTCTTTCCCTACACGACGCTCTTCCGATCT**NNNNNNacagtGAGCAGGCGCCCAAC
NGSl-AS1	**CTCGGCATTCCTGCTGAACCGCTCTTCCGATCT**NNNNNNatgtcATCAGAGGCAGGATTTCC

Table S7: Amino acid bias in Random AA library vs VWF73(NNK) library

The influence of nucleotide identity at the 3rd codon position on amino acid diversity is shown. Amino acids requiring G at this position are compared to amino acid that require T at this position and A, C, G, or T (NA) at this position. The abundance of T at this position within the VWF73(NNK) libraries (see Fig.S11 A,B) results in a biased amino acid diversity that likely explains the difference in amino acid content in this library compared to the random peptide library (NNK).

3rd nt	AA	NNK	VWF73-1	VWF73-2	Diff in NNK
G	M	2.32%	3.12%	3.01%	-0.75%
G	K	2.32%	2.01%	2.14%	0.24%
G	Q	2.87%	2.18%	2.08%	0.74%
G	W	3.27%	2.21%	2.10%	1.12%
G	E	4.77%	1.88%	1.84%	2.91%
T	F	1.82%	5.07%	5.98%	-3.71%
T	Y	1.95%	5.10%	5.45%	-3.33%
T	I	2.04%	4.98%	5.38%	-3.14%
T	C	3.51%	5.71%	5.60%	-2.14%
T	N	2.36%	4.21%	4.62%	-2.05%
T	H	2.24%	4.01%	3.89%	-1.71%
T	D	3.91%	3.32%	3.27%	0.61%
NA	L	7.06%	10.34%	10.43%	-3.33%
NA	S	8.59%	11.02%	11.03%	-2.44%
NA	T	4.51%	6.65%	6.46%	-2.04%
NA	P	5.78%	5.60%	5.42%	0.27%
NA	V	7.92%	6.81%	6.57%	1.23%
NA	A	8.14%	4.78%	4.41%	3.55%
NA	R	10.72%	6.09%	5.68%	4.84%
NA	G	13.89%	4.90%	4.60%	9.13%

Table S8: Recombinant clones for validation of phage display screens

Clone	Sequence
**LELYLS**	GACCGGGAGCAGGCGCCCAACTTGGAGTTGTATCTGTCTGGA AATCCTGCCTCTGATGAG
**IQLFLA**	GACCGGGAGCAGGCGCCCAACATTCAATTGTTTCTGGCTGGA AATCCTGCCTCTGATGAG
**RLRYFL**	GACCGGGAGCAGGCGCCCAACAGATTGAGATATTTTTTGGGA AATCCTGCCTCTGATGAG
**IMMFLG**	GACCGGGAGCAGGCGCCCAACATCATGATGTTTTTGGGAGGA AATCCTGCCTCTGATGAG
**LRYSSM**	GACCGGGAGCAGGCGCCCAACTTGCGTTATAGTAGTATGGGA AATCCTGCCTCTGATGAG
**NLQLIF**	GACCGGGAGCAGGCGCCCAACAATTTGCAATTGATCTTTGGA AATCCTGCCTCTGATGAG
**APPVDS**	GACCGGGAGCAGGCGCCCAACGCTCCACCGGTAGACAGTGGA AATCCTGCCTCTGATGAG
**SSWWMC**	GACCGGGAGCAGGCGCCCAACAGTAGTTGGTGGATGTGTGGA AATCCTGCCTCTGATGAG
**LGLEHS**	GACCGGGAGCAGGCGCCCAACCTTGGACTAGAACATAGTGGA AATCCTGCCTCTGATGAG
**LSVYGS**	GACCGGGAGCAGGCGCCCAACCTTAGTGTTTATGGAAGTGGA AATCCTGCCTCTGATGAG
